# DNA use in forensic human identification

**DOI:** 10.1080/07853890.2021.1893580

**Published:** 2021-09-28

**Authors:** Christine Keyser

**Affiliations:** University of Strasbourg, France

## Abstract

More than 99% of the DNA code is identical for all people. The remaining percentage is of interest to forensic scientists because of the variations in the DNA that exist between individuals and that allow to identify them. The purpose of the presentation is to give an overview of the strategies developed by the forensic experts to identify criminal offenders, to resolve unestablished paternity or identify remain of unknown soldier. Examples provided by our works on forensic or historical cases will illustrate this presentation.

